# LUCIDITY IN DEMENTIA: EMERGING CONCEPTS AND DATA

**DOI:** 10.1093/geroni/igac059.350

**Published:** 2022-12-20

**Authors:** Andrea Gilmore-Bykovskyi, Basil Eldadah

## Abstract

Episodes of lucidity among people with advanced Alzheimer’s disease and Alzheimer’s disease related dementias (AD/ADRD), reportedly evidenced by an unexpected return of meaningful communication or connection, are an area of growing interest. Event transient recovery of abilities in the setting of advanced AD/ADRD are potentially significant for caregivers and the person living with dementia; as well as for shaping our understanding of AD/ADRD more broadly. Despite the potential significance of these events, existing evidence is largely comprised of anecdotal reports and retrospective/case reports. Recently, the National Institute on Aging funded six studies to advance empiric studies on episodes of lucidity in advanced AD/ADRD. This symposium will provide an update on progress and findings across these funded studies. The first presentation focuses on acceptability and feasibility of a prospective observation study focused on characterizing potential observable indicators of episodes of lucidity in individuals with advanced AD/ADRD near end of life encompassing audiovisual observation, informant field interviews and case reviews. The second presentation will provide an overview of a 3-phase mixed methods study focused on development of preliminary types of lucid episodes. Collectively, presentations demonstrate a variety of approaches to investigating episodes of lucidity and shed light on emergent methods for operationalizing these events. Implications of various conceptualizations for EL and conceptual decisions across studies will be reviewed.

